# Risk Factors for Sepsis Based on Sepsis-3 Criteria after Orthotopic Liver Transplantation

**DOI:** 10.1155/2018/8703172

**Published:** 2018-06-20

**Authors:** Yanling Wang, Yu Gu, Fei Huang, Dezhao Liu, Zheng Zhang, Niman Zhou, Jiani Liang, Changyin Lu, Dongdong Yuan, Ziqing Hei

**Affiliations:** Department of Anesthesiology, The Third Affiliated Hospital of Sun Yat-sen University, Guangzhou City, Guangdong Province, China

## Abstract

Sepsis is a common complication of solid organ transplant procedures and, in particular, can affect the prognosis of orthotopic liver transplantation (OLT). This retrospective study determined the pre-, peri-, and postoperative risk factors for sepsis after OLT, using as reference the 2016 Third International Consensus Definitions for Sepsis and Septic Shock (Sepsis-3). Pre-, peri-, and postoperative clinical data of the sepsis-positive (*n* = 85) and sepsis-negative (*n* = 41) groups were analyzed for potential risk factors of OLT-related sepsis. The sepsis-positive patients had a significantly higher rate of dialysis (49.4%), longer time under mechanical ventilation (1.5 d), higher hospitalization costs (0.41 million RMB), and worse survival rate (68.5%), compared with the sepsis-negative patients (4.8%, 1 d, 0.30 million RMB, and 73.1%, resp.). The multivariate logistic analysis identified the following as risk factors for OLT-related sepsis: preoperative Child-Pugh grade C (OR 10.43; 95% CI 2.081–52.292; *P* = 0.004), preoperative hypercalcemia (OR 6.372; 95% CI 1.693–23.98; *P* = 0.006), and perioperative acidosis (OR 6.364; 95% CI 1.196–33.869; *P* = 0.030). Patients with preoperative Child-Pugh grade C, preoperative hypercalcemia, or perioperative acidosis are at higher risk for developing sepsis after OLT. When any of these problems occur, timely sepsis management should be planned.

## 1. Introduction

Liver transplantation (LT) is a life-saving therapy for patients with liver cancer and end-stage liver disease [1]. In the United States in the year 2015, 7127 LTs were performed [2]. The countries with the second and third highest rates of LT are China (>3500 LTs in 2005) and Brazil (1756 in 2015) [3, 4]. While the LT procedure has been standardized to a considerable extent, the management of postoperative complications remains a major challenge. In particular, LT patients are at elevated risk of developing sepsis, a systemic inflammation and immune system dysregulation [5–7]. Without proper and timely treatment, LT-related sepsis can result in longer hospital stays and even death. Identifying the risk factors of LT-related sepsis would facilitate its earlier recognition and management.

Many factors have been proposed as potential predictors of LT-related sepsis, including exhaled nitric oxide, aerobic capacity, plasma amino acid profile, and muscle wasting [8–10]. However, most of these risk factors concern only the postoperative period, and the value of these reports has been limited by the lack of a clear definition of postoperative sepsis.

In 2016, the Third International Consensus Definitions for Sepsis and Septic Shock (Sepsis-3) was released by the American Medical Association [11]. Sepsis-3 is defined as a life-threatening organ dysfunction caused by a dysregulated host response to infection, determined as a SOFA (sequential sepsis-related organ failure assessment) score ≥ 2 points [11]. Previous definitions of sepsis were based largely on scores related to systemic inflammatory response syndrome (SIRS), but the newly released Sepsis-3 guidelines emphasize the importance of the nonhomeostatic host response to infection and the SOFA score [12, 13]. Since its publication, the Sepsis-3 report has been cited 1673 times (as of October 6, 2017). Research results that utilize the Sepsis-3 criteria have a great value in clinical practice.

The present retrospective study applied the Sepsis-3 criteria to investigate the pre-, peri-, and postoperative risk factors of LT-related sepsis in 126 patients who underwent orthotopic liver transplantation (OLT).

## 2. Patients and Methods

### 2.1. Study Population

**Figure 1:**
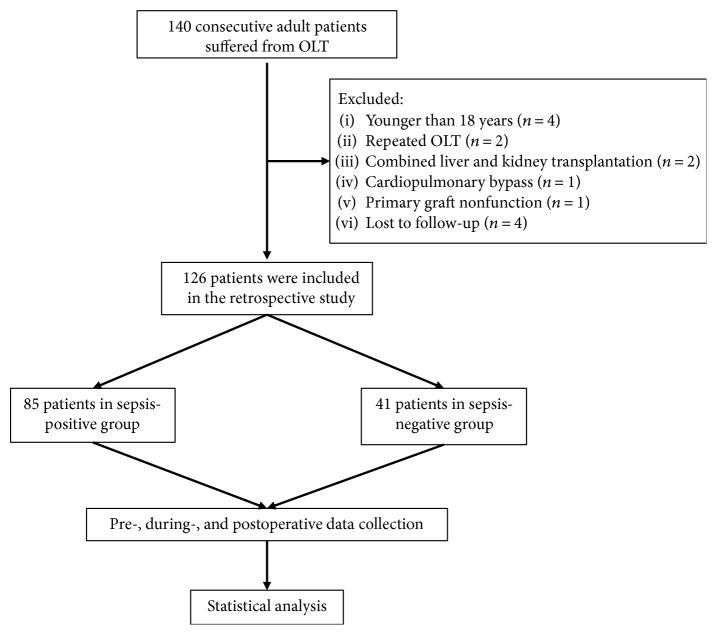
Study population and exclusion criteria in the current retrospective study.

The Research Ethics Committee at the Third Affiliated Hospital of Sun Yat-sen University, Guangzhou, China, reviewed and approved this retrospective study ([2015]2-190). After searching the medical records of the patients admitted from February 2012 to March 2015 at the Third Affiliated Hospital of Sun Yat-sen University, 140 consecutive OLT cases were obtained. Patients with any of the following were excluded from this analysis: aged <18 or >70 years (*n* = 4), repeat OLT (*n* = 2), combined liver and kidney transplantation (*n* = 2), cardiopulmonary bypass during OLT (*n* = 1), primary graft nonfunction (*n* = 1), or lost to follow-up (*n* = 4). Finally, 126 OLT cases were included and allotted to either a sepsis-positive group or sepsis-negative group, in which sepsis was diagnosed based on the newly released Sepsis-3 criteria (Figure 1).

Eleven patients with a SOFA score ≥ 2 points were identified as sepsis-positive [11]. The SOFA comprises scores relevant to the following organ systems and graded from 0 to 4 according to the degree of dysfunction or failure (see Vincent et al. 1998 for details) [14]: respiration (PaO_2_/FiO_2_ ratio), coagulation (platelets), the liver (bilirubin), cardiovascular (mean artery pressure), the central nervous system (Glasgow coma score), and renal (creatinine and urine output).

### 2.2. Data Collection

The following data were collected: gender; recipient age; body mass index (BMI); primary disease for OLT; history of previous surgeries, diabetes, and hypertension; hospitalization expenses; preoperative Child-Pugh class and model for end-stage liver disease (MELD) score; and preoperative infection. Preoperative laboratory tests included white blood cell count (WBC); serum creatinine (SCr); blood urea nitrogen (BUN); prothrombin time (PT); activated partial thromboplastin time (APTT); blood glucose; and serum levels of sodium, potassium, and calcium.

Perioperative data included the following: hepatic cold ischemia time; durations of anesthesia, OLT, and anhepatic phase; hemorrhage and blood transfusion volumes (red blood cell, plasma, and fresh frozen plasma); intraoperative fluid or albumin infusions; hypokalemia and acidosis; and usage of ulinastatin (Guangdong Techpool Bio-pharma, Guangzhou City, Guangdong Province, China).

Postoperative observations were duration of mechanical ventilation; time in intensive care unit (ICU); infection; and usage of alprostadil (Beijing Tide Pharmaceutical, Beijing, China), terlipressin (Hybio Pharmaceutical, Shenzhen City, Guangdong Province, China), and recombinant human interleukin 11 (rhIL-11; Qilu Pharmaceutical, Jinan City, Shandong Province, China).

### 2.3. Statistical Analysis

Continuous and categorical variable data are presented as mean ± standard deviation or number (percentage). The normality of the measurement data was tested using the one-sample Kolmogorov-Smirnov test. Abnormally distributed measurement data are shown as median and range. A 2-tailed independent sample *t*-test was used to compare normally distributed measurement data, and the Wilcoxon rank sum test was used to compare abnormally distributed measurement data. Pearson's chi-squared test was applied to analyze enumeration data (except for survival rate). Variables that were significantly different between the sepsis-positive and sepsis-negative groups were subjected to logistic regression for univariate and multivariate analyses. Significance was set at *P* < 0.05. All statistical analyses were performed using SPSS 23 (IBM, Chicago, IL) software.

## 3. Results

### 3.1. Incidence of Sepsis after OLT

Based on the Sepsis-3 criteria, 85 of the 126 OLT patients were diagnosed as sepsis. Detailed information with regard to the diagnosis of sepsis is listed in Supplemental Table 1 and Supplemental Excel File 1. Eleven of those sepsis patients developed septic shock. The major pathogens were Gram-negative bacteria and fungi (Table 1).

### 3.2. Prognosis of Patients with or without OLT-Related Sepsis

**Figure 2:**
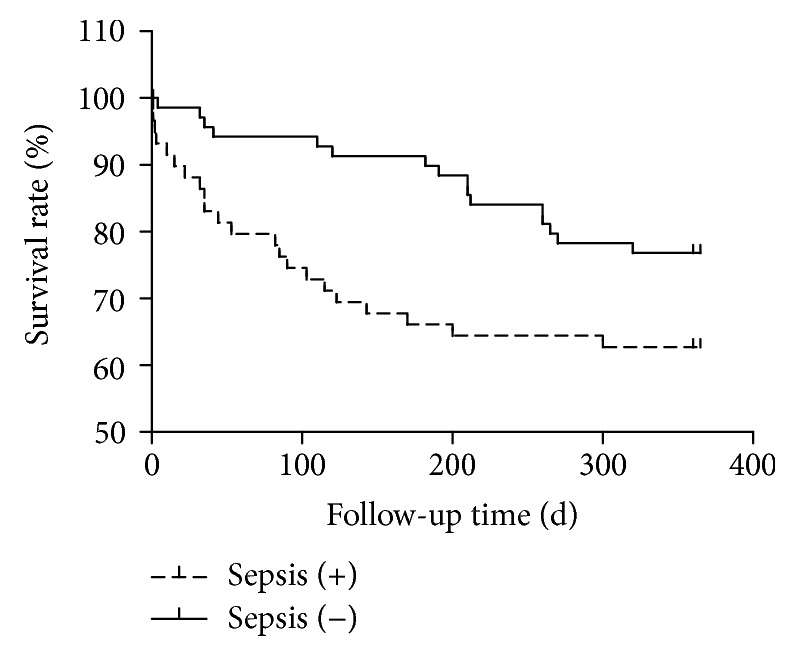
Survival rate and sepsis.

Compared with the sepsis-negative group (Table 2), a significantly higher percentage of the patients in the sepsis-positive group underwent dialysis after OLT (49.4% cf. 4.8%, *P* < 0.001), and the duration of mechanical ventilation was longer (1.5 d cf. 1 d, *P* = 0.022). Patients with sepsis had significantly worse survival rate than patients without sepsis (*P* = 0.0215) (Figure 2). The sepsis-positive patients also had higher hospitalization costs (0.41 million cf. 0.30 million, Chinese RMB; *P* < 0.001) (Table 2).

### 3.3. Preoperative Data Screening

The sepsis-positive and sepsis-negative groups were significantly different with regard to the following (Tables 3 and 4): gender ratio (*P* = 0.044), Child-Pugh grade (*P* = 0.014), MELD score (*P* = 0.049), and serum calcium concentration (*P* = 0.011). All of the 16 women in this study suffered sepsis. Compared with the sepsis-negative group, a significantly larger percentage of patients in the sepsis-positive group was Child-Pugh grade C, had a MELD score < 11, and experienced hypercalcemia.

### 3.4. Perioperative Data Screening

Compared with the sepsis-negative patients, during the OLT, the sepsis-positive group had significantly higher volumes of red blood cell infusion (*P* = 0.010) and fluid infusion (*P* = 0.049) (Table 5). The percentage of patients who evidenced perioperative acidosis was also significantly higher in the sepsis-positive patients (*P* = 0.017).

### 3.5. Postoperative Data Screening

None of the postoperative observations (albumin infusion volume, the incidence of pneumonia and intestinal infection, serum potassium level, and blood glucose level) were significantly different between the sepsis-positive and sepsis-negative groups (*P* > 0.05, see Table 6).

### 3.6. Risk Factors for Sepsis after OLT

During the initial data screening, 7 pre- and perioperative characteristics were identified as potential risk factors for sepsis: gender ratio; preoperative Child-Pugh stage, MELD score, and blood calcium level; and peri-operative red blood cell infusion and total fluid infusion, and acidosis. Since the 16 women in this study all experienced sepsis, the gender ratio was excluded from the subsequent logistic regression. The patients were stratified based on perioperative red blood cell infusion (≤2250 ml and >2250 ml).

The univariate analysis showed that the following preoperative features were risk factors for sepsis after OLT (Table 7): Child-Pugh grade C (*P* = 0.006) and hypercalcemia (*P* = 0.014). In addition, the univariate analysis showed that the following perioperative features were risk factors for sepsis after OLT: red blood cell transfusion > 2250 ml (*P* = 0.033) and acidosis (*P* = 0.029).

Results of the multivariate analysis indicated that the following were independent risk factors of sepsis after OLT (Table 8): preoperative Child-Pugh grade C (OR 10.43; 95% CI 2.081–52.292; *P* = 0.004), preoperative hypercalcemia (OR 6.372; 95% CI 1.693–23.98; *P* = 0.006), and perioperative acidosis (OR 6.364; 95% CI 1.196–33.869; *P* = 0.030).

## 4. Discussion

Sepsis is a common and serious postoperative problem in OLT patients. In this study, 67% of the patients developed sepsis after OLT, and the development of sepsis was associated with higher rates of dialysis, prolonged duration of mechanical ventilation, greater economic burden, and worse overall survival. Early recognition of sepsis would help enable timely treatment and benefit the patients physically and economically. Our results indicated that the following factors may predict sepsis after OLT: preoperative Child-Pugh class C, preoperative hypercalcemia, and perioperative acidosis. Thus, both pre- and perioperative management strategies are essential for controlling postoperative sepsis in OLT patients.

Of particular note is that, in the current study, the newly published Sepsis-3 criteria were applied for the diagnosis of postoperative sepsis and categorizing patients for the analysis. The Sepsis-3 criteria were developed with reference to the latest understanding of the pathobiology, management, and epidemiology of sepsis [11] and have been accepted as a more specific and sensitive definition when compared with previous sepsis definitions [14, 15]. Since Sepsis-3 utilizes the SOFA score but does not consider SIRS, a small portion of the previous sepsis-positive patients may be considered sepsis-negative, and some of the previous sepsis-negative may be considered sepsis-positive [16, 17]. Thus, the risk factors of OLT-related sepsis that were identified by previous sepsis definitions require reevaluation under the new Sepsis-3 definition.

Nemes et al. conducted a retrospective study with 199 consecutive OLT patients and found that the recipient Child-Pugh grade could be used as an independent risk factor for predicting sepsis [18]. While those researchers used a previous definition of sepsis, in the present study, we confirmed that the preoperative Child-Pugh grade was an independent risk factor for sepsis under the Sepsis-3 definition. In Yoshizumi et al., another study conducted under the previous sepsis definition, the preoperative MELD score (>15) and the duration of the OLT (>14 h) were identified as independent risk factors for postoperative bacterial sepsis [19]. However, in our current study which grouped patients based on Sepsis-3, the preoperative MELD score and the duration of OLT had no statistical value for predicting sepsis.

This is the first study to identify hypercalcemia as an independent risk factor for sepsis after OLT. In general, critical surgical illness is accompanied by low total calcium concentration, low ionized calcium concentration, or both [20]. However, Forster et al. reviewed 100 patients in the surgical ICU and found that 85 of them developed hypercalcemia after surgery [20]. Interestingly, in the same study, they reported that patients who developed hypercalcemia had significantly higher rates of shock, bacteremia, renal failure, and significantly more transfusions, antibiotics, dialysis, and parenteral nutrition usage, when compared with the patients without hypercalcemia [20]. These results suggest that preoperative hypercalcemia may be an important risk factor for severe illness, including sepsis, in the surgical ICU. Sugimoto et al. reported that sepsis was associated with higher levels of circulatory proinflammatory cytokines (particularly interleukin-6) and bone resorption, by promoting osteoclast activity and inhibiting osteoblast activity [21]. These features subsequently led to hypercalcemia. In the current study, preoperative hypercalcemia was identified as a predictor of post-OLT sepsis. Thus, timely monitoring and maintaining the serum calcium level would help improve the outcome of OLT.

The blood lactic acid level has increasingly been considered as an independent risk factor of sepsis severity [22, 23]. Tissue hypoxia and epinephrine-induced stimulation of aerobic glycolysis have been proposed as the main mechanisms that contribute to lactic acid accumulation during sepsis [20]. Severe lactic acidosis (blood pH < 7.2) is closely associated with high mortality rates in patients with sepsis [24]. Lee et al. reported that the lactate level 6 hours after early quantitative resuscitation is a strong predictor of a 28-day mortality in patients with sepsis or septic shock [25]. Marty et al. reported that lactate clearance during the first 24 hours could be used to predict a 28-day mortality rate in septic patients hospitalized in the surgical ICU [26]. In the present study, the perioperative, but not postoperative, blood lactic acid level showed statistical value for predicting sepsis after OLT.

The goal of the present study was to investigate the potential risk factors for OLT-related sepsis under the newly released Sepsis-3 criteria [11]. This study did not attempt to compare the differences between Sepsis-3 and the previous sepsis definitions.

While statistically valid risk factors were identified, this study has limitations. Most of the livers were donated from cardiac death donors. Additional work will be needed to verify the current findings with a larger sample scale and well-recorded follow-up.

## 5. Conclusions

This is the first report that identifies risk factors for OLT-related sepsis based on the newly released Sepsis-3 criteria. This study found that preoperative Child-Pugh grade C, preoperative hypercalcemia, and perioperative acidosis were independent risk factors for sepsis after OLT. Thus, for patients scheduled for OLT, recognizing the importance of preoperative Child-Pugh grade C, preoperative hypercalcemia, and perioperative acidosis should help to plan timely management and improve the prognosis of OLT.

## Figures and Tables

**Table 1:** The major pathogens for sepsis after orthotopic liver transplantation (OLT).

Pathogens	Percentage (%)
Gram-negative bacteria	29
*Acinetobacter baumannii*	10
*Klebsiella pneumoniae*	5
*Pseudomonas aeruginosa*	3
*Enterobacter cloacae*	2
*Stenotrophomonas maltophilia*	6
*Flavobacterium meningosepticum*	2
*Ralstonia pickettii*	1
Fungi	14
*Candida albicans*	5
*Candida glabrata*	3
*Candida guilliermondii*	2
*Trichosporon asahii Akagi*	1
Others	3

**Table 2:** Prognosis of patients with or without orthotopic liver transplantation- (OLT-) related sepsis (reported as *n* (%), unless otherwise noted).

	Sepsis-negative	Sepsis-positive	*P*
Subjects, *n*	41	85	—
Dialysis	2 (4.8)	42 (49.4)	<0.001
Reintubation/tracheostomy	3 (7.3)	16 (18.8)	0.334
Fiber bronchoscope suction	8 (19.5)	10 (11.7)	0.543
Medicines after OLT			
Alprostadil	19 (46.3)	21 (24.7)	0.052
Terlipressin	6 (14.6)	10 (11.8)	0.880
RhIL-11^a^	17 (41.5)	19 (22.4)	0.071
Duration of ICU^b^ stay, d	3 (2–5)	3.5 (2–10)	0.072
Mechanical ventilation, d	1 (1–1)	1.5 (1–3)	0.022
Returning to ICU	3 (7.3)	14 (16.5)	0.438
Hospitalization after OLT, d	35 (28–48)	44 (38–63)	0.066
Hospitalization costs, 10^6^ RMB^c^	0.30 (0.24–0.34)	0.41 (0.34–0.63)	<0.001

^a^RhIL-11: recombinant human interleukin 11; ^b^ICU: intensive care unit; ^c^RMB: Chinese renminbi.

**Table 3:** Preoperative clinical characteristics of patients with or without orthotopic liver transplantation- (OLT-) related sepsis (normally distributed data) (reported as *n* (%) unless otherwise noted).

	Sepsis-negative	Sepsis-positive	*P*
Age, y	44.44 ± 8.63	49.08 ± 10.92	0.067
Male/female	41/0	69/16	0.044
Portal hypertension	21 (51.2)	46 (54.1)	0.555
Hypertension	0	8 (9.4)	0.260
Serum albumin, g/l	32.69 ± 5.62	35.54 ± 6.08	0.155
Smoking	6 (14.6)	13 (15.3)	1.000
Drinking	8 (19.5)	13 (15.3)	0.886
Diabetes	10 (24.4)	18 (21.2)	0.813
Etiology			
Liver cirrhosis	14 (34.1)	27 (31.8)	0.099
Severe hepatitis	6 (14.6)	19 (22.4)	
Hepatic carcinoma	17 (41.5)	17 (20)	
Others	3 (7.3)	21 (24.7)	
Child-Pugh grade			
A	14 (34.1)	8 (9.4)	0.014
B	11 (26.8)	21 (24.7)	
C	16 (39.0)	56 (65.9)	
MELD^a^ score			
<11	9 (22.0)	35 (41.2)	0.049
11–18	19 (46.3)	24 (28.2)	
19–24	5 (12.2)	21 (24.7)	
≥25	8 (19.5)	5 (5.9)	
Serum potassium			
Normal	35 (85.4)	61 (71.8)	0.372
Hypokalemia	5 (12.2)	21 (24.7)	
Hyperkalemia	2 (4.8)	3 (3.5)	
Serum sodium			
Normal	25 (60.9)	56 (65.9)	0.694
Hyponatremia	16 (39.0)	29 (34.1)	
Serum calcium			
Normal	33 (80.5)	43 (50.6)	0.011
Hypercalcemia	8 (19.5)	42 (49.4)	
Blood glucose			
Normal	22 (53.7)	54 (63.5)	0.461
Hypoglycemia	11 (26.8)	13 (15.3)	
Hyperglycemia	8 (19.5)	18 (21.1)	
Pneumonia	17 (41.5)	27 (31.8)	0.372
Biliary tract infection	6 (14.6)	6 (7.1)	0.491
Other infection	10 (24.4)	13 (15.3)	0.578

^a^MELD: model for end-stage liver disease.

**Table 4:** Preoperative measurements of patients with or without orthotopic liver transplantation- (OLT-) related sepsis (abnormally distributed data).

	Sepsis-negative	Sepsis-positive	*P*
Subjects, *n*	41	85	—
White blood cell count, ×10^9^/l	10.13 (3.45–15.43)	5.17 (2.84–10.37)	0.061
Prothrombin time, s	21.80 (16.70–33.00)	26.80 (15.90–34.70)	0.754
Activated partial thromboplastin time, s	71.00 (46.00–76.00)	59.00 (41.00–71.40)	0.236
Serum creatinine, *μ*mol/l	80.00 (66.00–168.50)	84.00 (66.50–105.50)	0.406
Blood urea nitrogen, mmol/l	13.60 (4.53–31.56)	6.20 (3.88–12.61)	0.059

**Table 5:** Perioperative characteristics of patients with or without orthotopic liver transplantation- (OLT-) related sepsis.

	Sepsis-negative	Sepsis-positive	*P*
Cold ischemia, h	6.00 (6.00–8.00)	6.00 (5.25–6.75)	0.217
OLT, h	6.92 (6.11–8.42)	7.00 (6.07–8.33)	0.829
Anhepatic phase, min	52.00 (46.00–60.50)	52.00 (47.00–65.00)	0.728
Ulinastatin, million U	0.60 (0.53–0.65)	0.60 (0.3–0.63)	0.082
Albumin infusion, ml	200 (150–300)	250 (150–300)	0.365
Red blood cell transfusion, ml	1675 (750–2800)	2800 (1500–4400)	0.010
Plasma transfusion, l	3.00 (2.35–4.00)	3.40 (2.30–5.50)	0.286
Fresh frozen plasma transfusion, U	39.00 (22.50–47.50)	40.00 (30.00–50.00)	0.485
Fluid infusion, ml	6375 (5690–7300)	7700 (6253–10,120)	0.049
Bleeding volume, ml	2500 (1000–3450)	3000 (1800–6000)	0.113
Urine volume, ml	1600 (1300–2125)	1350 (763–2000)	0.081
Hypokalemia, *n* (%)	5 (12.2)	26 (30.6)	0.068
Acidosis, *n* (%)	3 (7.3)	27 (31.8)	0.017

**Table 6:** Postoperative clinical characteristics of patients with or without orthotopic liver transplantation- (OLT-) related sepsis (reported as *n* (%), unless otherwise noted).

	Sepsis-negative	Sepsis-positive	*P*
Subjects, *n*	41	85	—
Albumin infusion, ml	1700 (600–2450)	2100 (950–2900)	0.357
Pneumonia	29 (70.7)	53 (62.4)	0.720
Intestinal infection	13 (31.7)	18 (21.2)	0.328
Serum potassium			
Normal	29 (70.7)	37 (43.5)	0.060
Hypokalemia	11 (26.8)	46 (54.1)	
Hyperkalemia	2 (4.8)	2 (2.3)	
Blood glucose			
Normal	8 (19.5)	27 (31.8)	0.231
Abnormal^a^	33 (80.5)	58 (68.2)	

^a^All of the abnormal blood glucose patients were in hyperglycemia, except one patient was in hypoglycemia in the sepsis-positive group.

**Table 7:** Univariate logistic analysis for the incidence of sepsis after orthotopic liver transplantation (OLT) (reported as *n* (%), unless otherwise noted).

	B	SE	Wals	Sig.	Exp (B) 95% CI
Preoperative					
Child-Pugh grade				
A	—	—	7.734	0.021	—
B	1.207	0.729	2.743	0.098	3.343 (0.802–13.942)
C	1.841	0.663	7.705	0.006	6.3 (1.718–23.108)
MELD^a^ score					
<11	—	—	7.353	0.061	—
11–18	−1.076	0.602	3.198	0.074	0.341 (0.105–1.109)
19–24	0.167	0.789	0.045	0.832	1.182 (0.252–5.547)
≥25	1.81	0.863	4.395	0.116	0.164 (0.103–1.889)
Hypercalcemia	1.397	0.568	6.043	0.014	4.044 (1.327–12.323)
Perioperative					
RBC^b^ infusion (>2250 ml)	1.112	0.521	4.558	0.033	3.04 (1.095–8.436)
Fluid infusion	1.647	1.107	3.014	0.812	5.689 (1.283–35.687)
Acidosis	1.735	0.793	4.789	0.029	5.667 (1.18–26.793)

^a^MELD: model for end-stage liver disease; ^b^RBC: red blood cell.

**Table 8:** Multivariate logistic analysis for the incidence of sepsis after orthotopic liver transplantation (OLT).

		B	SE	Wals	Sig.	Exp (B) 95% CI
Preoperative Child-Pugh grade				
A	—	—	8.152	0.017	—
B	1.652	0.891	3.441	0.064	5.22 (0.911–29.915)
C	2.345	0.823	8.126	0.004	10.43 (2.081–52.292)
Hypercalcemia		1.852	0.676	7.5	0.006	6.372 (1.693–23.98)
Perioperative acidosis		1.851	0.853	4.706	0.03	6.364 (1.196–33.869)

## Supplementary Materials

Supplementary Materials Supplementary Table 1: diagnosis of the 85 sepsis patients according to Sepsis-3 criteria.
