# Supplementary material for: Risk Factors for Sepsis Based on Sepsis-3 Criteria after Orthotopic Liver Transplantation
Source: Mediators Inflamm. 2018 Jun 20;2018:8703172. doi: 10.1155/2018/8703172 (PMC6031077; doi:10.1155/2018/8703172)
Supplement: Supplementary Materials — Supplementary Table 1: diagnosis of the 85 sepsis patients according to Sepsis-3 criteria. [file 8703172.f1.zip › 8703172.f1/Supplemental-Table 1.doc]

Supplemental-Table 1. Diagnosis of the 85 sepsis patients according to Sepsis-3 criteria.

| **Case No.** | **Time (after OLT)** | **Infection (P-Positive) ^a^** | | | | | **CRP**  **(mg/L)** | **PCT**  **(ng/ml)** | **Respiratory system score ^b^** | **Nervous system score** | **Cardiovascu-lar system score** | **Liver function score** | **Coagulation system score** | **Kidney function score** | **SOFA**  **Score** | **Therapy ^c^** |
| --- | --- | --- | --- | --- | --- | --- | --- | --- | --- | --- | --- | --- | --- | --- | --- | --- |
|  |  | **Lung** | **Intestine** | **Urinary system** | **biliary tract** | **Blood** |  |  |  |  |  |  |  |  |  |  |
| **1** | 6d | P |  |  |  |  | 25.42 | 30.65 | 3 | 4 | 4 | 2 | 3 | 1 | 17 | MP+VY+MD+CF+GC |
| **2** | 3d | P |  |  |  |  | 21.76 | 15.59 | 2 | 2 | 0 | 2 | 0 | 1 | 7 | MP+VY+CF+GC |
| **3** | 2d | P |  |  |  |  | 34.07 | 86.98 | 2 | 0 | 1 | 1 | 0 | 3 | 7 | MP+VY+MD+GC |
| **4** | 5d |  | P |  |  |  | 39.7 | 5.59 | 1 | 1 | 4 | 2 | 2 | 0 | 10 | PT+MD+GC |
| **5** | 2d | P |  |  |  |  | 64.9 | 13.76 | 2 | 1 | 0 | 3 | 0 | 0 | 6 | PT+LF+VY+MD |
| **6** | 22h |  |  | P | P |  | 30.86 | 30.6 | 3 | 2 | 1 | 3 | 2 | 3 | 14 | MP+VY+MD+GC |
| **7** | 4d | P |  |  |  |  | 80.05 | 60.15 | 2 | 1 | 0 | 0 | 2 | 0 | 5 | PT+MD+CF+GC |
| **8** | 5d | P |  |  |  |  | 87.34 | 26.79 | 1 | 0 | 0 | 0 | 0 | 4 | 5 | PT+MD+GC |
| **9** | 2d | P |  |  |  |  | 35.91 | 10.74 | 1 | 0 | 0 | 2 | 2 | 0 | 5 | MP+MD+MS+GC |
| **10** | 3d | P |  |  |  |  | 37.05 | 7.42 | 1 | 0 | 1 | 0 | 1 | 1 | 4 | PT+MS+GC |
| **11** | 3d |  | P |  |  |  | 15.2 | 17.93 | 1 | 0 | 0 | 2 | 0 | 4 | 7 | MP+MD+VY+GC |
| **12** | 5d | P |  |  |  |  | 17.7 | 31.85 | 2 | 0 | 0 | 1 | 1 | 0 | 4 | PT+MD+CF+GC |
| **13** | 5d | P |  |  |  |  | 51.9 | 92.63 | 2 | 2 | 4 | 2 | 1 | 4 | 15 | IP+VY+MD+CF+GC |
| **14** | 6d |  | P |  |  |  | 28.8 | 2.82 | 1 | 0 | 0 | 3 | 0 | 0 | 4 | MP+VY+MD+CF+GC |
| **15** | 6d |  |  |  |  | P | 23.4 | 42.93 | 1 | 0 | 4 | 2 | 0 | 0 | 7 | PT+VY+CF+GC |
| **16** | 4d | P |  |  |  |  | 21.6 | 25.85 | 2 | 0 | 4 | 2 | 2 | 3 | 13 | MP+VY+CF+GC |
| **17** | 7d |  | P |  |  |  | 35.2 | 42.57 | 0 | 2 | 4 | 1 | 2 | 1 | 10 | MP+VY+MD+MS+GC |
| **18** | 3d |  |  |  |  | P | 55.88 | 5.07 | 0 | 2 | 0 | 2 | 0 | 1 | 5 | CSS+IP |
| **19** | 1d | P |  |  |  |  | 37.3 | 25.99 | 3 | 2 | 0 | 2 | 1 | 4 | 12 | PT+MD+CF+GC |
| **20** | 4d | P |  |  |  |  | 40.6 | 117.21 | 2 | 2 | 4 | 0 | 0 | 0 | 8 | MP+VY+MD+GC |
| **21** | 3d | P |  | P |  |  | 30.36 | 21.76 | 1 | 0 | 0 | 2 | 1 | 3 | 7 | PT+MD+MS+GC+EC |
| **22** | 5d |  | P |  |  |  | 70 | 21.31 | 1 | 0 | 0 | 3 | 3 | 0 | 7 | MP+MD+VY+CF+GC |
| **23** | 4d |  | P |  |  |  | 33.3 | 25.8 | 2 | 0 | 0 | 1 | 3 | 0 | 6 | PT+MD+MS+GC |
| **24** | 7d |  |  |  |  | P | 25.69 | 11.99 | 1 | 0 | 4 | 3 | 2 | 1 | 11 | PT+MD+LF+CF+VY+GC+EC |
| **25** | 10d | P |  |  |  |  | 47 | 44.34 | 2 | 2 | 1 | 4 | 3 | 3 | 15 | MP+MD+VY+CF+GC |
| **26** | 6d | P |  |  |  | P | 35 | 10.56 | 2 | 2 | 0 | 2 | 2 | 0 | 8 | MP+MD+CSS |
| **27** | 5d | P |  |  | P | P | 46.84 | 42.18 | 2 | 3 | 0 | 3 | 3 | 0 | 11 | MD+MP+VY+CF+CSS |
| **28** | 24h | P |  |  |  |  | 38.54 | 8.62 | 3 | 0 | 0 | 1 | 2 | 0 | 6 | PT+CSS+CF |
| **29** | 4d |  |  |  |  | P | 49.36 | 3.46 | 2 | 0 | 0 | 1 | 3 | 3 | 9 | PT+MD+CF+EC+GC |
| **30** | 6d | P |  |  |  |  | 25.64 | 4.63 | 3 | 2 | 4 | 3 | 2 | 3 | 17 | MP+CF+EC |
| **31** | 2d |  |  |  |  | P | 24.59 | 29.34 | 1 | 0 | 4 | 2 | 2 | 0 | 9 | MP+CF+MD+EC |
| **32** | 10d |  | P |  |  |  | 23.9 | 32.84 | 2 | 2 | 1 | 3 | 2 | 0 | 10 | PT+MD+MS+GC |
| **33** | 2d | P |  |  |  |  | 14.5 | 10.13 | 1 | 0 | 0 | 2 | 2 | 0 | 5 | MP+CF+EC |
| **34** | 3d |  | P |  |  |  | 23.28 | 7.07 | 1 | 0 | 1 | 4 | 2 | 0 | 8 | PT+MD+MS+GC |
| **35** | 4d |  | P |  |  |  | 51.84 | 23.1 | 0 | 3 | 2 | 2 | 2 | 3 | 12 | PT+IN+GC |
| **36** | 3d | P |  |  |  |  | 89.3 | 8.22 | 2 | 3 | 4 | 1 | 2 | 2 | 14 | MP+MD+VY+CF+GC |
| **37** | 2d | P |  |  |  | P | 14 | 8.2 | 2 | 2 | 4 | 4 | 4 | 1 | 17 | PT+IN+GC |
| **38** | 6d | P |  |  | P |  | 46.47 | 32.83 | 3 | 0 | 2 | 3 | 2 | 1 | 11 | CSS+MD+MS+EC |
| **39** | 7d |  | P |  |  |  | 43.21 | 5.79 | 1 | 0 | 2 | 3 | 2 | 3 | 11 | MP+CSS+VY+CF |
| **40** | 4d |  |  |  |  | P | 28.04 | 59.05 | 1 | 0 | 4 | 3 | 2 | 0 | 10 | CSS+IP |
| **41** | 2d | P |  |  |  |  | 14.3 | 7.7 | 2 | 0 | 0 | 3 | 1 | 0 | 6 | TP+MF+VY+VN |
| **42** | 3d | P |  |  |  |  | 14.2 | 28.04 | 3 | 3 | 3 | 2 | 3 | 1 | 15 | IP+MD+VY |
| **43** | 5d | P |  |  |  |  | 89.9 | 11.54 | 2 | 1 | 0 | 2 | 3 | 0 | 8 | MP+CF+MD+GC+CSS+TC |
| **44** | 5d | P |  |  |  |  | 22.34 | 5.87 | 1 | 3 | 0 | 2 | 2 | 2 | 10 | MP+CF+VY+MD |
| **45** | 4d | P |  |  |  |  | 74.5 | 26.46 | 2 | 0 | 3 | 3 | 3 | 3 | 14 | MP+TC+TP+VN+CSS |
| **46** | 7d | P |  | P |  |  | 255.586.85 | 141.06 | 3 | 4 | 2 | 2 | 3 | 3 | 17 | MP+CF+MD+GC+CSS+TC |
| **47** | 16h |  |  |  | P |  | 69.3 | 87.03 | 4 | 3 | 1 | 4 | 3 | 0 | 15 | PT+MD+MS+GC |
| **48** | 4d | P |  |  |  |  | 60 | 48.51 | 2 | 2 | 0 | 3 | 3 | 1 | 11 | MP+CF+GC+MD |
| **49** | 5d | P |  |  | P |  | 28 | 7.13 | 2 | 0 | 4 | 4 | 3 | 2 | 15 | MP+CF+GC+MD |
| **50** | 6d | P |  |  |  |  | 70.7 | 42.55 | 3 | 2 | 3 | 2 | 3 | 1 | 14 | IP+CF+VY+GC |
| **51** | 6d | P |  |  |  |  | 20.12 | 16.89 | 1 | 2 | 3 | 2 | 2 | 2 | 12 | MP+CF+GC+MD |
| **52** | 14d | P |  |  |  |  | 15.2 | 5.97 | 3 | 0 | 3 | 4 | 3 | 4 | 17 | MP+CF+MD+GC |
| **53** | 8d | P |  |  |  |  | 10.7 | 101.87 | 3 | 2 | 4 | 3 | 3 | 4 | 19 | IP+CF+MD+VY |
| **54** | 9d | P |  |  |  |  | 138 | 60.39 | 1 | 0 | 0 | 3 | 2 | 4 | 10 | MP+TP+CF+MD+GC |
| **55** | 4d |  |  |  |  | P | 22.5 | 2.53 | 0 | 0 | 0 | 1 | 0 | 0 | 1 | MP+TP+CF+MD+GC |
| **56** | 6d | P |  |  |  |  | 10.23 | 6.52 | 1 | 3 | 0 | 3 | 3 | 4 | 14 | TC+TP+MP+VN+CSS |
| **57** | 8d | P |  |  |  |  | 29.01 | 19.56 | 2 | 0 | 0 | 4 | 2 | 2 | 10 | TC+TP+MP+VN+CSS |
| **58** | 5d | P |  |  |  |  | 56.52 | 19.11 | 3 | 3 | 3 | 2 | 4 | 0 | 15 | TC+MP+VN+CSS |
| **59** | 3d | P |  |  |  |  | 27.6 | 8.25 | 2 | 2 | 0 | 0 | 3 | 0 | 7 | MP+VN+CF+GC |
| **60** | 6d |  | P |  |  |  | 22.6 | 5.12 | 1 | 0 | 0 | 2 | 4 | 1 | 8 | MP+VY+MD |
| **61** | 4d |  | P |  |  |  | 16.14 | 8.97 | 1 | 0 | 0 | 2 | 3 | 1 | 7 | CSS+TC |
| **62** | 9d | P |  |  |  |  | 167.1 | 52.09 | 3 | 4 | 3 | 3 | 3 | 2 | 18 | MP+CF+GC+MD |
| **63** | 3d |  | P |  |  |  | 106.3 | 8.21 | 0 | 2 | 0 | 2 | 1 | 1 | 6 | CF+IP+EC |
| **64** | 12d |  |  | P | P |  | 20.7 | 26.45 | 2 | 0 | 0 | 4 | 2 | 4 | 12 | MP+CF+EC+MS |
| **65** | 10d |  |  |  | P | P | 97.8 | 2.15 | 1 | 0 | 4 | 3 | 3 | 2 | 13 | MP+VY+MD+GC |
| **66** | 9d |  |  |  | P | P | 30.3 | 7.22 | 1 | 0 | 0 | 2 | 1 | 0 | 4 | CSS+MD+MS+EC |
| **67** | 8d |  |  |  | P |  | 3.15 | 30.4 | 1 | 0 | 1 | 4 | 3 | 0 | 9 | VY+CF+GC+PT |
| **68** | 10d | P |  |  |  |  | 41.01 | 11.23 | 1 | 0 | 1 | 2 | 4 | 0 | 8 | MP+CF+GC+MD+VY |
| **69** | 7d | P |  |  |  |  | 10.8 | 49.81 | 1 | 3 | 1 | 2 | 3 | 0 | 10 | VY+MP+CF+GC |
| **70** | 10d |  | P |  |  |  | 71.5 | 3.64 | 0 | 1 | 1 | 1 | 2 | 0 | 5 | IP+CF+GC |
| **71** | 4d | P |  |  |  |  | 34.8 | 20.15 | 2 | 0 | 1 | 2 | 2 | 1 | 8 | VY+MD+MP+CF |
| **72** | 6d | P |  |  |  |  | 21.13 | 8.64 | 2 | 0 | 4 | 0 | 3 | 2 | 11 | MP+CF+GC+MD |
| **73** | 7d |  | P |  |  |  | 56.7 | 34.39 | 1 | 0 | 1 | 1 | 3 | 1 | 7 | PT+MD+MS+GC |
| **74** | 5d |  | P |  |  |  | 25.3 | 38.09 | 1 | 0 | 1 | 2 | 2 | 0 | 6 | CSS+MS+MD+GC |
| **75** | 6d | P |  |  |  |  | 86.73 | 58.22 | 2 | 0 | 4 | 0 | 0 | 2 | 8 | MP+CF+GC+MD+VY |
| **76** | 4d | P |  |  |  |  | 13.98 | 23.07 | 2 | 4 | 4 | 3 | 3 | 1 | 17 | MP+CF+MD+VY |
| **77** | 7d | P |  |  |  |  | 56.01 | 33.6 | 1 | 0 | 0 | 2 | 2 | 1 | 6 | MP+GC+MD+VY+CF |
| **78** | 3d | P |  |  |  |  | 68.47 | 7.03 | 1 | 0 | 1 | 3 | 2 | 1 | 8 | MP+GC+MD+VY+CF |
| **79** | 4d |  |  |  |  | P | 40.49 | 20.21 | 1 | 0 | 0 | 2 | 2 | 0 | 5 | IP+MP+VY+CF |
| **80** | 3d | P |  |  |  |  | 47.4 | 9.91 | 2 | 0 | 1 | 2 | 2 | 1 | 8 | MP+CF+GC+MD+VY |
| **81** | 3d | P |  |  |  |  | 80.4 | 39.7 | 2 | 0 | 1 | 2 | 2 | 0 | 7 | EC+VY+VN+IP+CF+CSS |
| **82** | 4d |  | P |  |  |  | 11.1 | 55.75 | 1 | 0 | 1 | 2 | 2 | 1 | 7 | GC+MP+VY+CZS |
| **83** | 9d |  | P |  | P |  | 27.93 | 56.9 | 2 | 0 | 1 | 4 | 2 | 1 | 10 | PT+MD+MS+GC |
| **84** | 5d | P |  |  |  |  | 31.46 | 27.32 | 2 | 4 | 2 | 4 | 0 | 2 | 14 | VY+MD+CF+PT |
| **85** | 3d | P |  |  |  |  | 24.83 | 50.64 | 3 | 2 | 1 | 2 | 2 | 3 | 13 | MP+VY |

^a^ Pulmonary infection was diagnosed by sputum culture and lung X-ray. Intestine, urinary system, and blood infection were diagnosed by stool culture, urine culture, and blood culture, respectively. Biliary tract infection was diagnosed by ultrasound and laboratory examination.

^b^ PaO2/FiO2, Glasgow score, Bilirubin level, Platelet, and creatinine level for 85 sepsis patients were listed in the sheet named “Supplemental-Excel File 1”.

^c^ Meropenem, MP; Metronidazole, MD; Vancomycin, VY; Caspofungin, CF;Ganciclovir, GC; Piperacillin-Tazobactam, PT; Levofloxacin, LF;

Micafungin Sodium, MS; Imipenem, IP; Cefoperazone Sodium Sulbactam Sodium, CSS; Entecavir, EC; Itraconazole, IN; Tazobactam and Piperacillin, TP; Moxifloxacin, MF; Voriconazole, VN; Tigecycline, TC; Teicoplanin, TP; Ceftizoxime sodium, CZS.
